# Deep-sea amphipods around cobalt-rich ferromanganese crusts: Taxonomic diversity and selection of candidate species for connectivity analysis

**DOI:** 10.1371/journal.pone.0228483

**Published:** 2020-02-06

**Authors:** Akira Iguchi, Miyuki Nishijima, Yuki Yoshioka, Aika Miyagi, Ryuichi Miwa, Yuichiro Tanaka, Shogo Kato, Takaaki Matsui, Yoshiaki Igarashi, Nobuyuki Okamoto, Atsushi Suzuki

**Affiliations:** 1 Geological Survey of Japan, National Institute of Advanced Industrial Science and Technology (AIST), Tsukuba, Ibaraki, Japan; 2 Department of Bioresources Engineering, National Institute of Technology, Okinawa College, Nago-City, Okinawa, Japan; 3 Kaiyo Engineering Co., Ltd., Taito-ku, Tokyo, Japan; 4 Japan Oil, Gas and Metals National Corporation (JOGMEC), Minato-ku, Tokyo, Japan; National Cheng Kung University, TAIWAN

## Abstract

The aim of this study was to select a candidate deep-sea amphipod species suitable for connectivity analyses in areas around cobalt-rich ferromanganese crusts (CRCs). We applied DNA barcoding based on the mitochondrial protein-coding gene, cytochrome c oxidase subunit I (*COI*), to specimens collected from the Xufu Guyot (the JA06 Seamount) off southeastern Minami-Torishima Island in the North Pacific, where CRCs are distributed. We used baited traps to collect 37 specimens. Comparison of *COI* sequences with public reference databases (GenBank, BOLD) showed that almost all of the specimens belonged to the superfamily Lysianassoidea, which is known to be ubiquitous in deep-sea areas. In a molecular taxonomic analysis of these sequences, we detected 11 clades. One of these clades (group 9) composed of 18 sequences and was identified by DNA barcoding as a putative species belonging to *Abyssorchomene*, which has been reported from the New Hebrides Trench in the South Pacific. We considered this species to be a candidate for connectivity analysis and analyzed its genome by restriction site–associated DNA sequencing. The results showed that the genetic variation in this species is adequate for analyzing connectivity patterns in CRC areas in the future.

## Introduction

Cobalt-rich ferromanganese crusts (CRCs) in deep-sea areas are attracting international attention as possible future sources of marine minerals. CRCs occur on the summits and slopes of seamounts (e.g., large flat-topped guyots), which are characterized by a unique fauna [1]. The International Seabed Authority (ISA) strongly recommends that contractors for exploration and exploitation of CRCs carry out baseline surveys for marine fauna and environmental conditions following ISA guidelines [2]. Specific requirements include information about biological communities. in particular is strongly required. The marine benthos around CRCs includes the animal communities that are most likely to be disturbed by the mining of marine minerals because benthic organisms live at or near the seafloor surface. Environmental impact assessments of the population dynamics and resilience of the deep-sea marine benthos around CRCs are thus essential before deep-sea mining can be initiated [1]. These assessments require evaluation of the genetic diversity and connectivity of the benthic populations. DNA markers for obtaining such information about populations have been broadly applied to many animal taxa [3], including the deep-sea benthos [4,5].

One difficulty in performing population-level studies in deep-sea areas is that many of the species are unknown or undescribed; few taxonomic studies are available because sampling sites are difficult to access [6]. In the former case, DNA barcoding is a promising approach for selecting putative species for connectivity analysis under these circumstances [7,8]. Whether DNA barcoding can be used to distinguish a putative species depends on the targeted DNA regions and the evolutionary history of the targeted taxon (e.g., the degree of lineage sorting). A fragment of the mitochondrial cytochrome c oxidase subunit I gene (*COI*) has been used as a standard “taxon barcode” for many animal groups [7,9], and this locus is also known to have relatively high resolution at inter- and intraspecies levels [8]. In addition, a huge number of *COI* sequences have been registered in public databases [9] and can be used to infer which species might be similar to an unknown or undescribed species.

Among deep-sea benthic taxa, the order Amphipoda (Crustacea: Malacostraca) is a major group and plays important roles in the deep-sea ecosystem. Furthermore, amphipods can be easily collected by using simple baited traps [10]. Thus, amphipod species in deep-sea areas might be good candidates for investigating connectivity patterns in deep-sea areas including those where CRCs are located. However, deep-sea amphipods include many cryptic species [10]. In this study, we applied DNA barcoding based on *COI* to deep-sea amphipods randomly collected from the JA06 seamount. CRCs are widely distributed around this seamount, which is situated near Minami-Torishima Island in the North Pacific [11], and the International Seabed Authority (ISA) has licensed the area for mineral exploration and exploitation. In addition, we performed a restriction site–associated DNA (RAD) sequencing analysis by applying the ezRAD technique, which is a simple and cost-effective method [12,13] for generating and examining genetic variation. This approach was applied to deep-sea candidate amphipod species selected by DNA barcoding.

## Materials and methods

### Sampling, DNA extraction and library preparation

We used amphipod specimens collected with baited traps (shrimp pot, conger tube), including baited traps mounted on the Edokko Mark I benthic observation system (Okamoto Glass Co., Ltd.), at three sites (Table 1). A total of 37 specimens were used for this study. Specimens were preserved by immersion in absolute ethanol followed by freezing, and genomic DNA was extracted from the pleopods of each specimen according to the manufactures protocol of the Qiagen DNeasy Blood and Tissue Kit. DNA was also extracted from some specimens preserved in formalin. Partial sequences of the *COI* region were determined by the method described in the ISA Technical Study No. 13 [14]. To prepare a library for ezRAD, we used a Qubit Fluorometer (ThermoFisher, Waltham, USA) to measure the DNA concentration in each DNA sample. A library for RAD-Seq was prepared following the ezRAD protocol [12, 13] using the Illumina TruSeq library preparation kit. A bioanalyzer was used to check the quality, and paired-end reads (2 x 75 base pairs) were obtained. We also sequenced the genome of one specimen using a Nextera XT DNA Sample Prep Kit (Illumina) and obtained paired-end (2 x 300 base pairs) reads. These reads were obtained using the Illumina Miseq sequencer following the manufacturer’s protocol.

**Table 1:** Summary of samples used in this study.

Sampling sites	Latitude	Longitude	Depth	Sampling methods	Number of specimens
JA06-T01	19°31.99'N	157°50.80'E	1,327 m	Baited traps (shrimp pot, conger tube)	3
JA06-T02	19°32.07'N	158°00.19'E	1,311 m	Baited traps (shrimp pot, conger tube)	15
JA06-B05	19°22.53'N	157°52.97'E	3,818 m	Baited traps (Edokko Mark I)	19

### Bioinformatics

**Fig 1:**
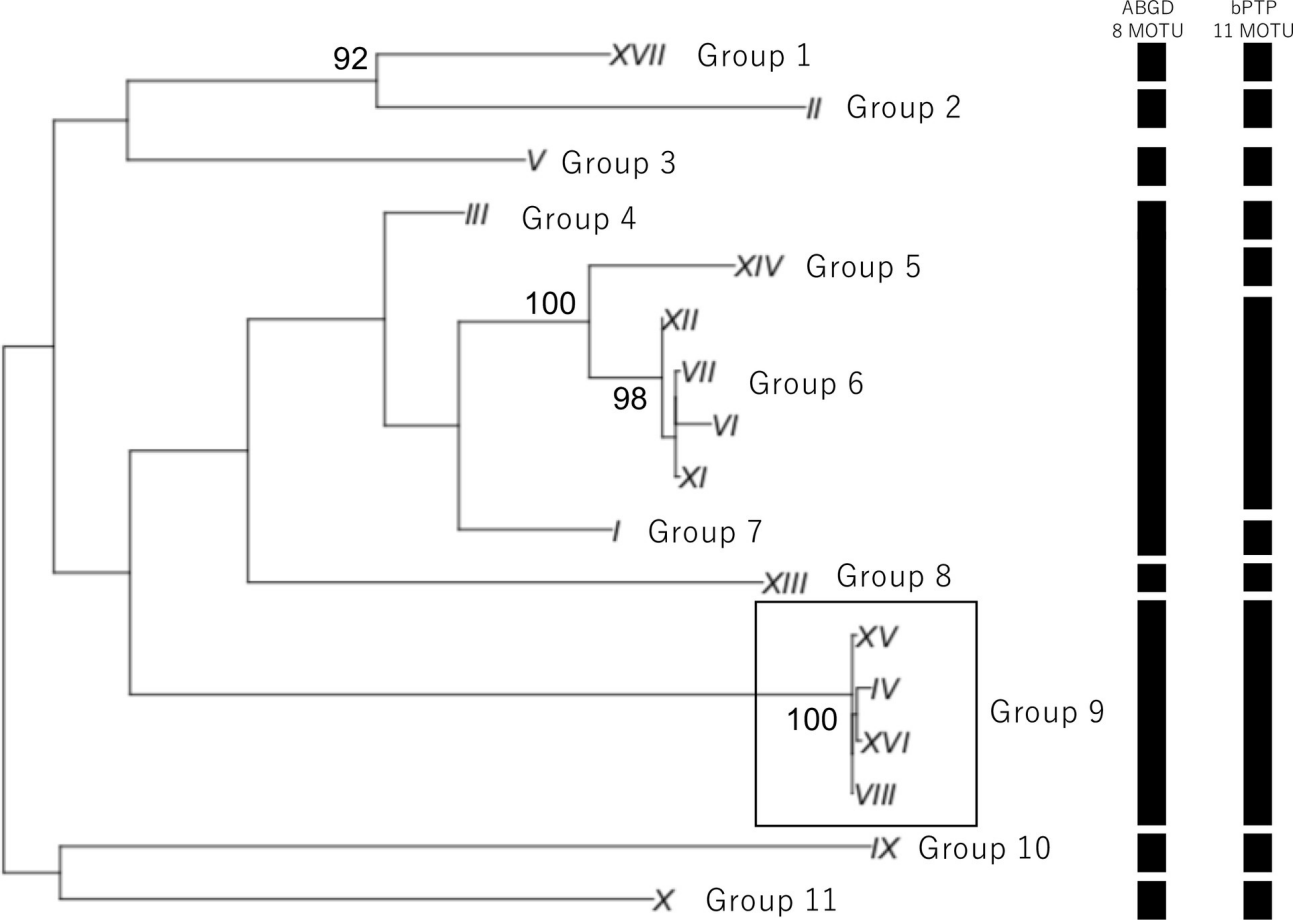
Molecular tree of the 17 amphipod species haplotypes obtained in this study (maximum likelihood method based on GTR + I + G model). Numbers indicate bootstrap values (1,000 replicates). One clade (Group 9, in the box) is composed of haplotypes inferred to represent a putative species.

**Fig 2:**
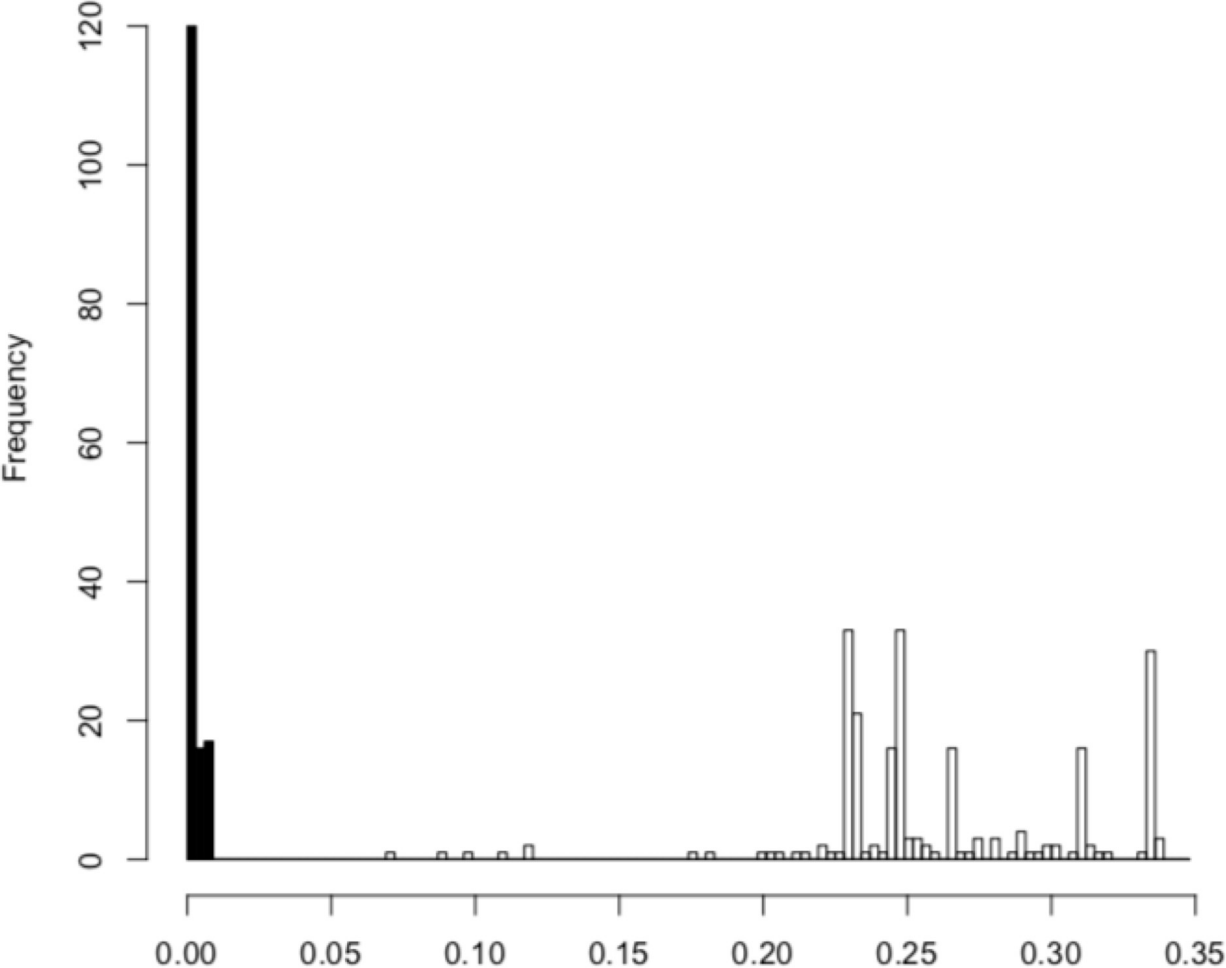
Histogram of intragroup and intergroup genetic distances. Closed bars show intra group distances of the 18 sequences inferred to represent a single species. Open bars show intergroup distances.

**Fig 3:**
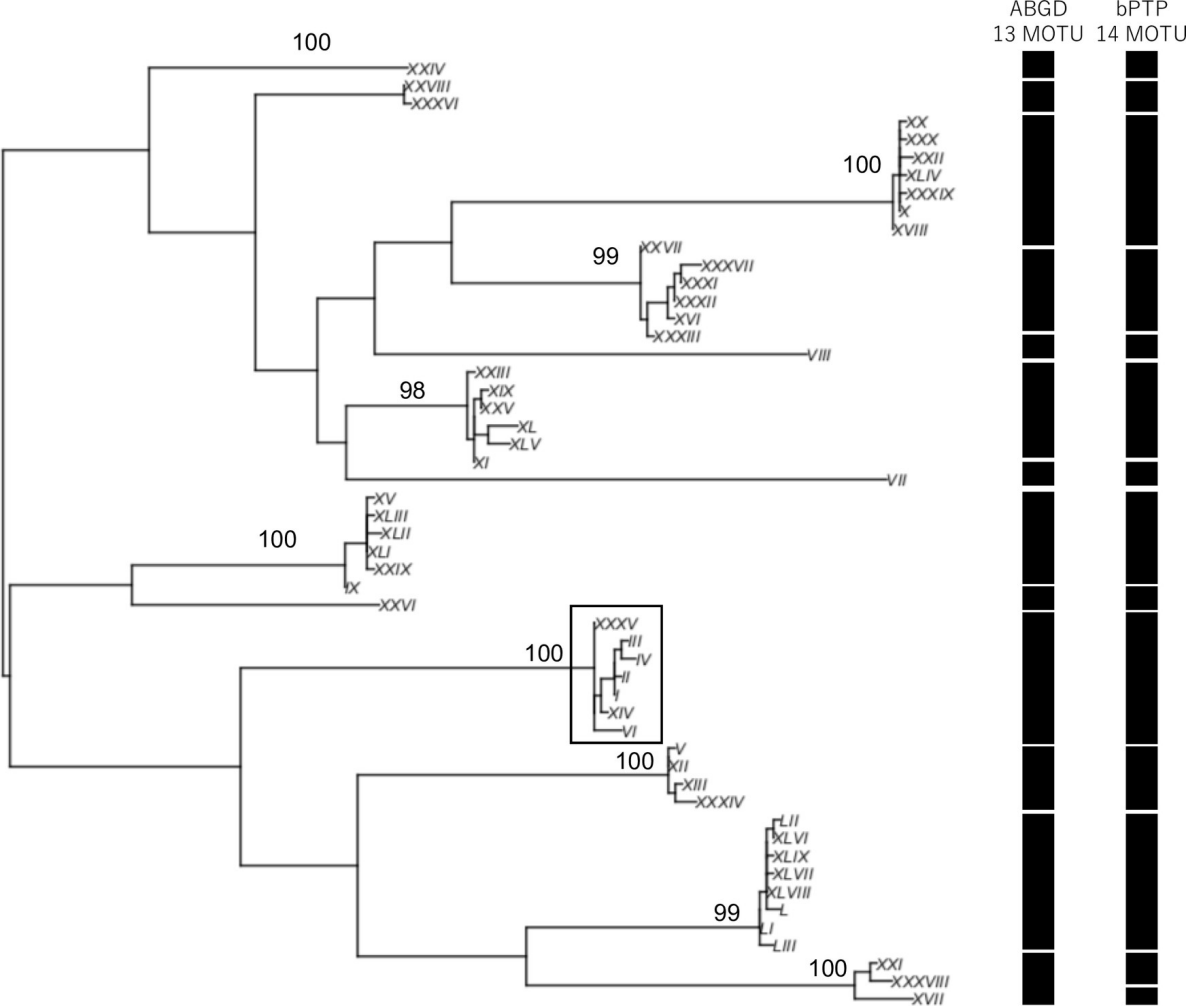
Molecular tree of 53 haplotypes belonging to genus *Abyssorchomene* (maximum likelihood method based on GTR + I + G model). Numbers indicate bootstrap values (1,000 replicates). One clade (in the box) is composed of haplotypes inferred to represent a single species.

We inferred 17 haplotypes from the partial *COI* sequences, and then we performed a BLASTN search (e-value cut-off: 1e^-5^) against the NCBI nt database (July, 2018) to identify possible neighboring species. We also used BOLD database (www.boldsystems.org) to infer the species of these 17 haplotypes. Then a maximum likelihood method was used to generate a tree for visualizing the genetic diversity of the amphipod species (Fig 1). For the analysis, we used a GTR + I + G model and 1,000 bootstrap replicates to estimate the statistical support for each clade. To evaluate the “barcoding gap” between intraspecific variation and interspecific/interclade divergence [8], we used the Kimura two-parameter model [15] when calculating genetic distances (Fig 2) based on representative haplotypes of the 11 groups in Fig 1 (all haplotypes excluding XI, VI, and VII which are very similar to XII) and on all sequences of Group 9 (Fig 1), a putative species that was inferred to belonging to the genus *Abyssorchomene* on the basis of a BLASTN search. From the DNA Data Bank of Japan (DDBJ) database, we downloaded all *COI* sequences of *Abyssorchomene* and constructed a haplotype network using the downloaded sequences along with the *Abyssorchomene* sequences obtained in this study. We also generated a tree via the maximum likelihood method using the 53 haplotypes inferred from all *Abyssorchomene* sequences (Fig 3). We used the GTR + I + G model; clade support was evaluated by 1,000 bootstrap replicates. These analyses were performed with the R software package [16] and related packages (ape, pegas, phangorn, seqinr; [17–20]). To identify molecular operational taxonomic units (MOTUs), we applied the automatic barcode gap discovery [ABGD; 21] and Bayesian implementation of the Poisson Tree Process [bPTP; 22] methods. In the ABGD, we used the Kimura two-parameter model [15] and the relative gap width (X) for X = 1.5 an X = 1 in Figs 1 and 3, respectively because only one clade was supported in the latter case with X = 1.5. In the bPTP, we constructed neighbor-joining trees and performed the analysis with the default settings in the web version (http://species.h-its.org/ptp/; MCMC generations: 100,000; thinning: 100; burn-in: 0.1). We constructed a haplotype network of seven haplotypes of *Abyssorchomene* sp. using the TCS network algorithm [23] in PopART v. 1.7 [24]. All sequence data have been deposited in the DDBJ database (accession nos. LC484978–LC485014).

The obtained FASTQ files of high-throughput sequencing data were filtered by using cutadapt version 1.9.1 software [25], and the reads with poor-quality bases (Q < 20) and those with lengths < 40 base pairs (bp) were discarded. Genome sequences of one *Abyssorchomene* sp. were assembled by using fastq-join in the ea-utils package [26]. Redundant sequences were removed by using the CD-HIT-EST program [27]. We selected sequences longer than 400 bp by using Seqkit version 0.9.3 [28] and then used these sequences as genome data for *Abyssorchomene* sp. To facilitate the mapping of short reads obtained by ezRAD, we processed the FASTQ files and prepared 25-bp-long sequences in FASTA files. We used the bowtie2 program (default setting) to align the FASTA sequences obtained by using ezRAD with the *Abyssorchomene* sp. genomic data [29]. We then used Stacks software (programs: pstacks (default setting), cstacks (-b 1 -p 4 -n 5), sstacks (-b 1 -p 4), and genotypes (-b 1)) [30] to identify single nucleotide polymorphisms (SNPs) in the obtained SAM files. Short-read data have been deposited in the DDBJ data base (accession nos. DRA008512 and DRA008890). Using the catalog files made by Stacks, we prepared a NEXUS file that included SNP loci among 10 individuals for subsequent analyses. We also used a maximum likelihood method to generate a tree with RAxML ver. 8.2.7 software [31]. For the analysis, we used the GTR-GAMMA model and 1000 bootstrap replicates to estimate the clade confidence levels.

## Results and discussion

We applied DNA barcoding methods to deep-sea amphipods collected from CRCs and identified 17 haplotypes from 37 individual sequences (S1 Table). The BLASTN search indicated that almost all species belonged to the superfamily Lysianassoidea, which is ubiquitous in deep-sea areas ([10]; S2 Table). Several clades molecularly delimited via ABGD and bPTP were supported by high bootstrap values (Figs 1 and S1; Groups 1–11). Among these clades, Group 9 composed of four haplotypes (XV, VIII, XVI, and IV). These four haplotypes were similar to *Abyssorchomene* sp. sequences in the NCBI nt database (S2 Table). In addition, a significant difference between intragroup and intergroup genetic distances was also confirmed (“barcoding gap”; Mann–Whitney U test, *p* < 0.01; Fig 2). ABGD detected 8 and 13 MOTUs among the haplotypes in Figs 1 and 3, respectively. Eleven and 13 MOTUs were detected by bPTP among the haplotypes in Figs 1 and 3, respectively. Both methods supported the Group 9 as a single MOTU. We therefore selected this putative species as a candidate for the subsequent analysis because a relatively larger number of specimens was available.

**Fig 4:**
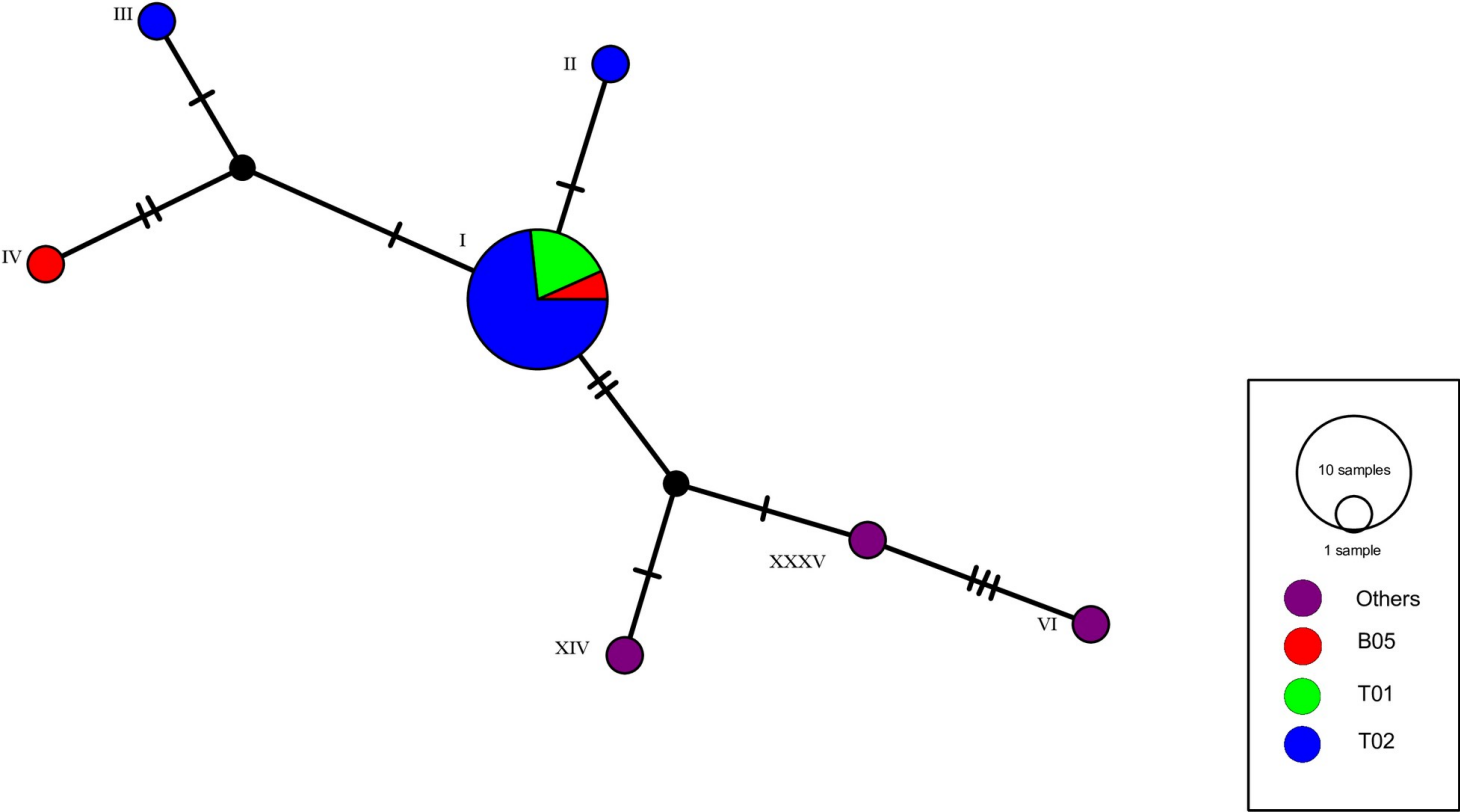
Haplotype network constructed from 7 haplotypes of *Abyssorchomene* sp. Greek numbers indicate haplotypes in Fig 3 and S3 Table.

We downloaded 71 *Abyssorchomene* sequences registered in the DDBJ database. Among these, we excluded two short sequences from our analyses. As a result, we used a total of 87 sequences: 69 downloaded sequences and 18 sequences obtained in this study via the analysis described above and inferred to be *Abyssorchomene* sp. From these sequences, 53 haplotypes were detected (S3 Table). Molecular analysis of these 53 haplotypes yielded several clades, one of which was composed of seven haplotypes inferred to represent a single *Abyssorchomene* sp. (Fig 3). MOTUs obtained by the ABGD and bPTP methods also supported this clade of *Abyssorchomene* sp. (Fig 3). The inclusion in this clade of the *Abyssorchomene* sp. sequences reported from the New Hebrides Trench in the South Pacific suggested that this *Abyssorchomene* sp. is widely distributed in both the North and South Pacific Oceans. The constructed haplotype network of this *Abyssorchomene* sp. showed a network structure that included one main haplotype and several others (Fig 4). We therefore selected this *Abyssorchomene* sp. as a candidate species for connectivity analysis of CRC areas in wide areas of the Pacific.

**Fig 5:**
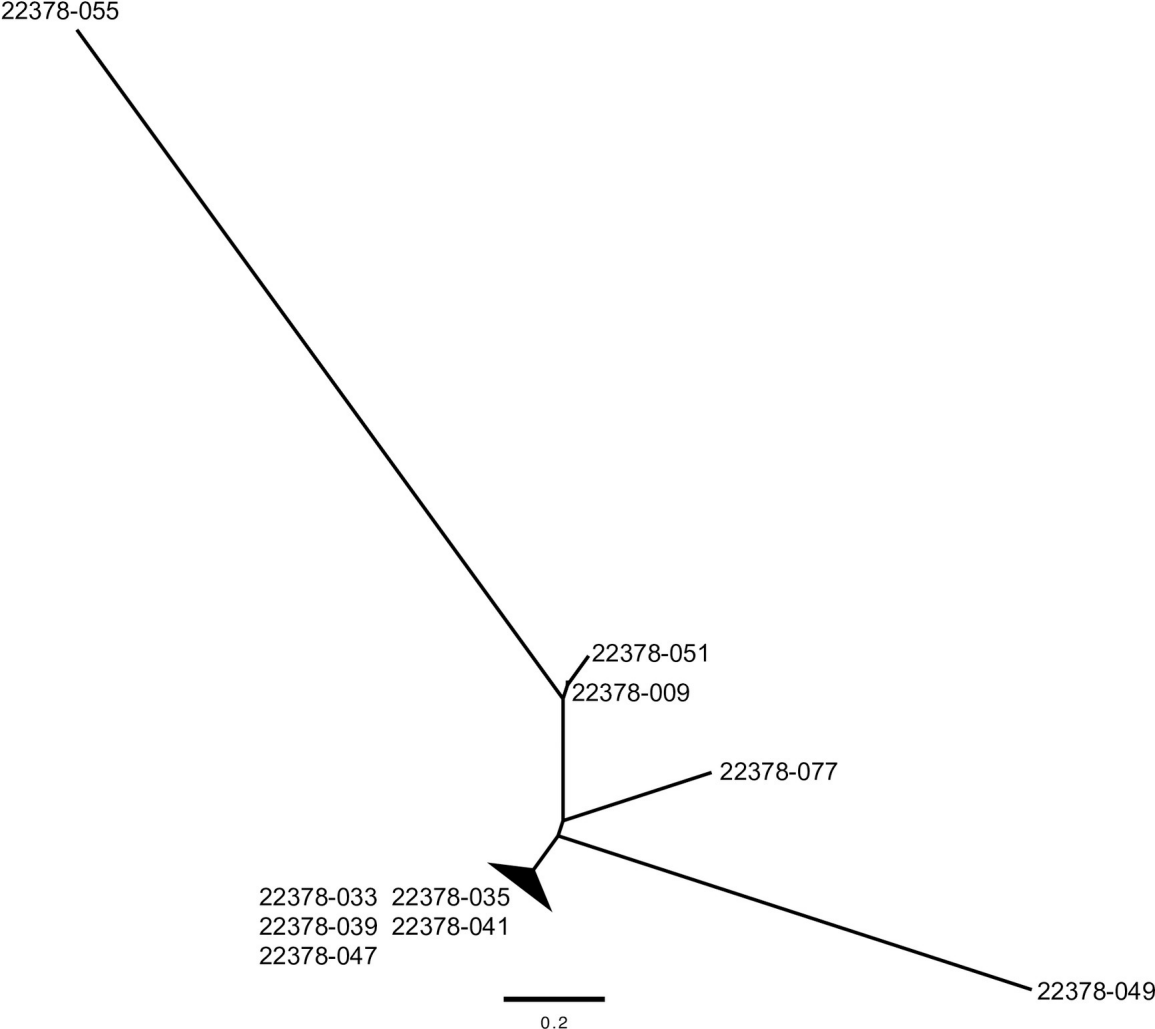
Molecular tree of 10 individuals inferred to belonging to a single *Abyssorchomene* sp (maximum likelihood method based on GTR-GAMMA model and 1,000 bootstrap replicates).

To evaluate the genetic variation of this candidate species in more detail, we performed an ezRAD analysis. The ezRAD libraries yielded on average 0.82 million 150-bp reads per individuals (S4 Table). Among these, we used the sequence data from 10 individuals (S4 Table), because after processing the data, we had obtained enough reads from these individuals for our analysis. We detected 75,265 SNPs among these 10 individuals. From the SNP polymorphisms, we inferred that all of these individuals were genetically distinct (Fig 5); this result implies that populations of this species are maintained mainly by sexual reproduction. Amphipod genera *Paralicella*, *Abyssorchomene*, and *Eurythenes* are reported to be cosmopolitan taxa widely distributed in abyssal zones [32], and several microsatellite markers have been developed for *Paralicella tenuipes* [33,34]. Considering the position of the *Abyssorchomene* sp. based on the tree of the *COI* region and the amount of polymorphism found by the ezRAD analysis, we infer that this species is also an appropriate species for use in future connectivity analyses in CRC areas. Considering the small number of specimens used in this study, an increased number of specimens would likely identify more candidate amphipod species for future connectivity analyses that would also enable us to use α and β diversity measures to evaluate community structures of deep-sea amphipods among CRC areas.

In conclusion, we succeeded in selecting a candidate amphipod species for understanding connectivity patterns around CRC areas. Connectivity among deep-sea organisms is now a matter of concern, especially in areas that are candidates for mining of marine minerals [4,5]. However, few genetic studies that would make it possible to generalize connectivity patterns have been conducted in deep-sea areas [35]. The evaluation of connectivity among mining areas is necessary before mining begins, and several connectivity analysis studies have been carried out in hydrothermal vent fields [36–38]. Nevertheless, information about connectivity in CRC areas is still lacking. The strategy as presented in this study is expected to facilitate the visualization of connectivity patterns around CRCs in deep-sea areas based on biological data. Knowledge of these patterns is essential not only for understanding the formation and maintenance of biodiversity, but also for establishing marine protected areas to minimize the effect of deep-sea mining of CRCs on the deep-sea ecosystem.

## Supporting information

S1 Fig Molecular tree based on partial sequences of the cytochrome-oxidase I gene (605 bp) of the amphipods collected from the JA06 seamount and neighboring species. The tree was constructed by MEGA6 [41] based on genetic distances of Kimura 2-parameter model [40] and Neighbor-Joining method [39]. Bootstrap values (percentages of 1,000 replications) >70% are shown at the respective selected branches. Bar means 0.05 substitutions per site. Greek numbers indicate 17 haplotypes.
(PDF)

S1 Table Haplotype compositions of the *COI* sequences used to construct Fig 1. (XLSX)

S2 Table Results of the BLASTN search against the nt database and BOLD (*COI* FULL database) using the *COI* sequences obtained in this study. (XLSX)

S3 Table Haplotype compositions of the *COI* sequences used to construct Fig 3. (XLSX)

S4 Table Details of the ezRAD analysis. Sample names in bold type were used in our final analysis.
(XLSX)
