# Supplementary figures and images for: Deep-sea amphipods around cobalt-rich ferromanganese crusts: Taxonomic diversity and selection of candidate species for connectivity analysis
Source: PLoS One. 2020 Feb 6;15(2):e0228483. doi: 10.1371/journal.pone.0228483 (PMC7004558; doi:10.1371/journal.pone.0228483)

Fig. S1

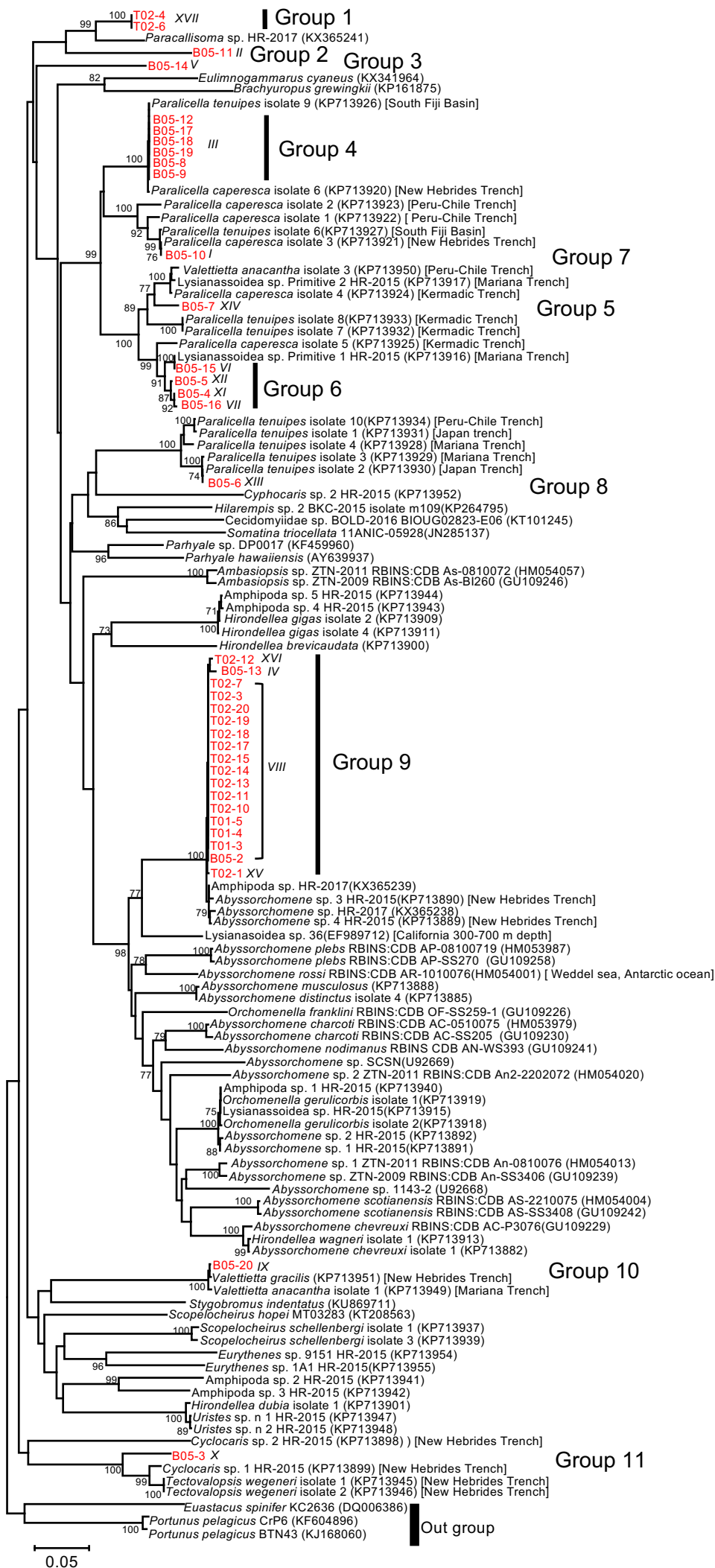

Supplement: S1 Fig — The tree was constructed by MEGA6 [41] based on genetic distances of Kimura 2-parameter model [40] and Neighbor-Joining method [39]. Bootstrap values (percentages of 1,000 replications) >70% are shown at the respective selected branches. Bar means 0.05 substitutions per site. Greek numbers indicate 17 haplotypes. (PDF) [file pone.0228483.s001.pdf]
